# Proliferation of macrophages due to the inhibition of inducible nitric oxide synthesis by oxidized low-density lipoproteins

**DOI:** 10.17179/excli2015-151

**Published:** 2015-03-20

**Authors:** Monika Brunner, Miriam Gruber, Diethart Schmid, Halina Baran, Thomas Moeslinger

**Affiliations:** 1 Institute for Physiology, Section for Vegetative Physiology, CEPP, Medical University Vienna, Schwarzspanierstraße 17, 1090 Vienna, Austria

**Keywords:** inducible nitric oxide synthase, lipoproteins, macrophages, atherosclerosis

## Abstract

Oxidized low-density lipoprotein (ox-LDL) is assumed to be a major causal agent in hypercholesteraemia-induced atherosclerosis. Because the proliferation of lipid-loaden macrophages within atherosclerotic lesions has been described, we investigated the dependence of macrophage proliferation on the inhibition of inducible nitric oxide synthase (iNOS) by hypochlorite oxidized LDL. Ox-LDL induces a dose dependent inhibition of inducible nitric oxide synthesis in lipopolysaccharide-interferon stimulated mouse macrophages (J774.A1) with concomitant macrophage proliferation as assayed by cell counting, tritiated-thymidine incorporation and measurement of cell protein. Native LDL did not influence macrophage proliferation and inducible nitric oxide synthesis. iNOS protein and mRNA was reduced by HOCl-oxidized LDL (0-40 µg/ml) as revealed by immunoblotting and competitive semiquantitative PCR. Macrophage proliferation was increased by the addition of the iNOS inhibitor L-NAME. The addition of ox-LDL to L-NAME containing incubations induced no further statistically significant increase in cell number. Nitric oxide donors decreased ox-LDL induced macrophage proliferation and nitric oxide scavengers restored macrophage proliferation to the initial values achieved by ox-LDL. The decrease of cytosolic DNA fragments in stimulated macrophages incubated with ox-LDL demonstrates that the proliferative actions of ox-LDL are associated with a decrease of NO-induced apoptosis. Our data show that inhibition of iNOS dependent nitric oxide production caused by hypochlorite oxidized LDL enhances macrophage proliferation. This might be a key event in the pathogenesis of atherosclerotic lesions.

## Introduction

Atherosclerosis is characterized by the deposition of lipids and the appearance of macrophages in the arterial wall. Oxidized LDL (ox-LDL) is assumed to be a major causal agent in hypercholesteraemia-induced atherosclerosis (Turunen et al., 2014[39]). LDL can undergo oxidative modification when incubated in vitro with endothelial cells, smooth muscle cells, or macrophages by the action of reactive oxygen intermediates, macrophage derived myeloperoxidase, or 15-lipoxygenase activity in endothelial cells. Oxidative modification of LDL involves peroxidation of lipids, an increase in the negative charge of the lipoprotein particles, conversion of phosphatidyl-choline to lysophosphatidyl-choline, and an alteration of epitopes on apolipoprotein B, resulting in its recognition and uptake by scavenger receptors on macrophages (Park, 2014[33]). As these receptors are not down-regulated by the intracellular cholesterol level, the macrophages transform to foam cells (Witztum, 1990[41]). Oxidatively modified LDL is present in macrophage foam cells of human atherosclerotic lesions (Ylä-Herttuala et al., 1989[48]). The dose- and time-dependent inhibition of inducible nitric oxide synthesis by oxidized LDL has been shown using mouse macrophages activated with IFN-gamma and/or LPS (Huang et al., 1999[24]; Yang et al., 1994[46]). Innate immune reactions have been described for atherosclerotic lesions. LDL is involved in vascular inflammatory processes, and approaches to new therapies are currently developed based on these concepts of the involvement of the immune system in atherosclerosis (Hansson and Hermannson, 2011[19]). 

Nitric oxide is synthesized from L-arginine by the L-arginine-nitric oxide pathway (Palmer et al., 1988[32]). A family of enzymes, termed the nitric oxide synthases (NOS), catalyzes the formation of nitric oxide and citrulline from L-arginine, O_2_, and NADPH (Marletta, 1993[30]). The inducible isoform of NOS (NOS-2 or iNOS, EC 1.14.13.39) generates large amounts of NO over a prolonged period of time through a Ca^2+^ independent pathway (Xie et al., 1992[43]). Inducible NOS expression has been observed in many cells, including murine macrophages (Hibbs et al., 1988[22]). NO inhibits proliferation of vascular smooth muscle cells, mesangial cells, cancer cells, and fibroblasts (Cheng et al., 2014[8]). 

Because the local proliferation of macrophages has been revealed as a key event in atherosclerosis (Robbins et al., 2013[35]; Rosenfeld and Ross, 1990[36]), and in situ hybridisation confirmed the presence of iNOS in macrophages, foam cells, and vascular smooth muscle cells of atherosclerotic vessels (Buttery et al., 1996[7]), we investigated the correlation between the inhibition of inducible nitric oxide synthesis by oxidatively modified LDL and macrophage proliferation. 

## Materials and Methods

### Materials 

[^3^H]L-arginine (68 Ci/mmol) was supplied by Amersham Corp., Arlington Heights, IL. [^3^H]thymidine (20 Ci/mmol) was obtained from ICN Pharmaceuticals, Costa Mesa, CA. Recombinant mouse gamma interferon (IFN-gamma) was purchased from Gibco BRL, Gaithersburg, MD. 2,2'-(Hydroxynitrosohydrazino) bis-etanamine (NOC 18) and 2-(4-Carboxyphenyl)-4,4,5,5-tetramethylimidazoline-1-oxyl-3-oxide (carboxy-PTIO) were obtained from Dojindo Laboratories, Tokyo, JP. Rabbit anti-iNOS polyclonal antibody and purified iNOS protein was supplied by Calbiochem, San Diego, CA. Cell culture materials, Escherichia coli lipopolysaccharide serotype 055:B5 (LPS) and all other chemicals were obtained from Sigma Chemical Co., St. Louis, MO.

### Cell culture 

The mouse monocyte/macrophage cell line J774.A1 (ATCC TIB 67) was cultured in Dulbecco´s Modified Eagle´s Medium (DMEM) supplemented with 10 % FBS, 25 mM HEPES, 2 mM Glutamine, 100 U penicillin/ml and 100 µg streptomycin/ml at 37 °C, 5 % CO_2_, and 95 % humidity. Cells were studied between passages 7-30. Prior to the experiments cells were cultured for 24 hours with lipoprotein deficient serum (LPDS). Thereafter, cells were seeded in 24-well dishes at a density of 2 x 10^5^ cells/well, stimulated by the addition of IFN-gamma (10 U/ml) and LPS (1 µg/ml), and incubated either with or without the indicated lipoproteins for 24 hours. 

### Nitrite analysis 

Nitrite was determined spectrophotometrically by using the Griess reagent (0.5 % sulfanilic acid, 0.002 % N-1-naphtyl-ethylenediamine dihydrochloride, 14 % glacial acetic acid) in supernatants. Absorbance was measured at 550 nm with baseline correction at 650 nm and nitrite concentration was determined using sodium nitrite as a standard (Green et al., 1982[17]). 

### Measurement of citrulline synthesis as a marker of NO production 

iNOS activity was determined by the conversion of arginine to citrulline. Citrulline was measured by a radiometric method as described previously (Bredt and Snyder, 1989[6]). IFN-gamma/LPS stimulated J774.A1 mouse macrophages were incubated at 37 °C with [^3^H]L-arginine (0.5 µCi/ml) for 15 minutes. The reaction was stopped by washing cells with cold phosphate-buffered saline. To separate [^3^H]L-citrulline from [^3^H]L-arginine, Dowex resin (AG 50W-X8, sodium form, Bio-Rad Lab., Richmond, CA, USA) was added to the cell lysates. The resin was allowed to settle for 15 minutes. Aliquots of the supernatants were used for liquid scintillation counting. [^3^H]L-citrulline formation was expressed as counts per minute (cpm) per mg cell protein above basal levels measured in unstimulated macro-phages. 

### Preparation and oxidation of LDL 

LDL (relative density 1.019 to 1.063 g/ml) was isolated from human plasma by sequential ultracentrifugation and was flotated through a potassium bromide solution of d=1.063 g/ml. After centrifugation LDL was filtered (0.45 µm pore size) and used immediately for further modification. LDL was transferred into PBS containing 100 µM EDTA by gel filtration through a size exclusion column (Bio-Rad Econo-Pac 10DG). The preparation of hypochlorite oxidized LDL was performed as described previously (Arnhold et al., 1991[3]) with minor modifications. The molar excess of hypochlorite over LDL was in the range of 400-600. After allowing the hypochlorite reagent to react with LDL for 15 min on ice, LDL was again passed over a size exclusion column (Bio-Rad 10DG) equilibrated with PBS containing 100 µM EDTA to remove excess reagent. Agarose gel electrophoresis of hypochlorite (HOCl) ox-LDL particles revealed an about twofold increase of the relative electrophoretic mobility. For the preparation of lipoprotein deficient serum (LPDS) fetal calf serum (Sigma) was depleted of lipoproteins by flotation at 100 000g with a density of 1.21 g/ml for 48 hours. After flotation the lipoprotein deficient serum was excessively dialyzed against saline and sterile filtered (0.22 µm pore size). 

### Tritiated thymidine incorporation 

Macrophage growth was assayed by the incorporation of [^3^H]thymidine (20 Ci/ mmol) into cellular DNA. [^3^H]thymidine (1 µCi/well) was added directly to the culture medium for the last two hours of an incubation. The medium was aspirated and the cells were washed with 500 µl ice-cold phosphate-buffered saline on ice before the addition of 500 µl methanol. Cell DNA was precipitated by adding 500 µl of 10 % trichloroacetic acid. Precipitates were lysed with 200 µl of 300 mM sodium hydroxide containing 1 % sodium dodecyl sulphate. Samples were aspirated and subjected to liquid scintillation counting. 

### Arginine transport 

Arginine uptake by macrophages was measured by adding [^3^H]L-arginine (68 Ci/mmol, 0.2 µCi/well) directly to the medium after a 24 hours incubation with or without ox-LDL as indicated. After 60 minutes the medium was aspirated and the cells were washed twice with 500 µl ice-cold phosphate-buffered saline. Cells were lysed with 200 µl of 300 mM sodium hydroxide containing 1 % sodium dodecyl sulphate. Samples were aspirated and subjected to liquid scintillation counting. 

### Cell number determination 

To determine the number of J774.A1 cells during culture, the number of adherent cells was counted within standard-sized areas (0.25 mm^2^) in each of three wells by inverted phase-contrast microscopy. 

### Cell protein 

Protein was determined according to the method of Bradford (Bradford, 1976[5]) using bovine serum albumin as standard.

### Western blotting for inducible nitric oxide synthase 

Cells were lysed in ice-cold buffer containing 25 mM monosodium phosphate (pH 7.4), 75 mM NaCl, 5 mM EDTA, 1 % Triton X-100, 100 µg/ml phenylmethylsulfonylfluoride, 10 µg/ml antipain, 10 µg/ml leupeptin, 10 µg/ml pepstatin, 20 µg/ml aprotinin, and 10 µg/ml trypsin inhibitor and centrifuged at 50 000g for 20 minutes at 4 °C. The cytosolic proteins (12 µg per lane) were separated by 8 % SDS-polyacrylamide gel electrophoresis. Proteins were transferred to nitrocellulose filters, and then immunoblotted with a rabbit anti-iNOS polyclonal antibody at a 1:1000 dilution. Anti-rabbit horseradish peroxidase-conjugated antibody was used as a secondary antibody at a dilution of 1:2500. The blots were detected with the enhanced chemiluminescence method and exposed to photographic film. 

### Semiquantitative competitive RT-PCR 

Total RNA was isolated using the guanidinium thiocyanate method (Chomczynski and Sacchi, 1987[9]). To determine the RNA concentration the absorption at 260, 280, and 320 nm was measured photometrically (UV/VIS Spectrophotometer Lambda 2, Perkin-Elmer, Norwalk, CT). Single-stranded cDNA synthesis was carried out on 2 µg of total RNA primed with oligo(dT)_12-18 _(Pharmacia, Freiburg, Germany) using murine leukemia virus reverse transcriptase (MMLV-RT; MBI Fermentas, Vilnius, Lithuania) at 37 °C for 60 min. Reactions were stopped by heating for 5 min at 70 °C. iNOS cDNA was subjected to DNA amplification by PCR using 0.5 units of Taq DNA polymerase (MBI) with oligonucleotide primers complementary to murine iNOS cDNA (MWG-Biotec, Ebersberg, Germany) at a final concentration of 0.25 µM. Oligonucleotide primers against beta-actin were used as the competitor at a concentration of 0.03 µM. Reaction mixtures were subjected to the following conditions in a PE 2400 DNA thermal cycler (Perkin-Elmer): denaturing at 94 °C for 30 seconds, annealing at 55 °C for 35 seconds, and extension at 72 °C for 35 seconds. After 32 cycles, the reaction mixture was cooled down to 4 °C. The primers for iNOS were 5´-CTA AGA GTC ACC AAA ATG GCT CCC-3´ (sense) and 5´-ACC AGA GGC AGC ACA TCA AAG C-3´ (antisense). The expected product length was 775 bp. The following primers for the “housekeeping gene” beta-actin were used: 5´-ATG GTG GGA ATG GGT CAG AAG GAC-3´(sense) and 5´-CTC TTT GAT GTC ACG CAC GAT TTC-3´ (antisense). The expected product length was 513 bp. All PCR-reactions were in linear range. Final PCR products were separated on a 1.2 % agarose gel and detected by ethidium bromide staining. Semiquantitative estimation was done by comparing mRNA expression of iNOS to beta-actin represented by the amount of the PCR product formed. 

### DNA fragmentation assay 

Cytosolic DNA fragments were quantified by a cell death detection ELISA (Boehringer Mannheim, Mannheim, Germany). The assay is based on the sandwich-enzyme-immunoassay principle using mouse monoclonal antibodies directed against histone-associated-DNA-fragments. Cells were washed with phosphate buffered saline and lysed with ELISA buffer. The lysates were centrifuged at 200 g for 10 min. The histone-associated-DNA-fragments of the supernatant were linked to the biotinylated anti-histone antibody bound to the streptavidin-coated microtiter plate as described by the manufacturer. The DNA-part of the nucleosomes was detected by peroxidase labelled anti-DNA antibody. The amount of histone-associated-DNA-fragments was quantified spectrophotometrically with 2,2´-azino-di(3-ethylbenzthiazolin-sulfonate) as substrate. Samples were read at 405 nm and 492 nm on a Perkin-Elmer Lambda 2 Spectrophotometer.

### Data analysis 

Each experimental result as shown in the figures is the mean ± SD for at least three measurements. When SD is not displayed, it is smaller than the size of the symbol. Statistical analyses were performed by use of ANOVA followed by Student´s *t-*tests for unpaired data. Statistical significance was defined as *P *< 0.05.

## Results

### Inhibition of inducible nitric oxide synthesis by ox-LDL 

**Figure 1:**
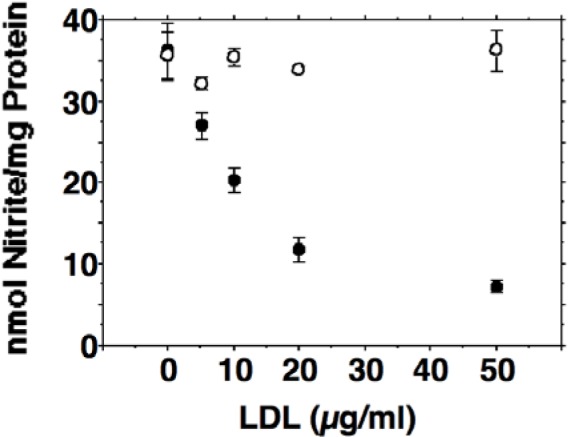
Dose-dependent inhibition of nitric oxide production by HOCl oxidized low-density lipoprotein (ox-LDL) in J774.A1 macrophages. Cell monolayers were preincubated with DMEM containing 10 % lipoprotein-deficient serum for 24 hours. Thereafter, cells were activated with 10 U/ml IFN-gamma and 1 µg/ml LPS and incubated with increasing amounts (0-50 µg/ml f.c.) of oxidized (• ) or native (o) LDL as indicated for 24 hours. After 24 hours the culture media were collected and assayed for nitrite as described in METHODS. Each data point shows the mean of triplicate measurements. Error bars show the standard deviation. *P* was < 0.01 or smaller for any ox-LDL containing incubations when compared to controls without ox-LDL. Differences between the incubations containing native LDL were not statistically significant.

Activated J774.A1 cells released large amounts of nitrite into the culture medium (36 ± 2.9 nmol nitrite per mg protein within 24 hours). Incubation of activated J774.A1 cells with oxidized LDL was associated with a dose dependent reduction in NO production (Figure 1(Fig. 1)). Incubations with native LDL revealed no inhibition of NO synthesis. To confirm our results that inducible nitric oxide synthesis is reduced by oxidized LDL, iNOS activity was determined by the conversion of arginine to citrulline described in the METHODS section. Citrulline formation was reduced by ox-LDL in a dose dependent manner from 380 ± 47.1 cpm/mg protein for control incubations of activated J774.A1 macrophages without ox-LDL to 206 ± 20.1 cpm/mg protein for 10 µg/ml ox-LDL, 77.1 ± 80.8 cpm/mg protein for 20 µg/ml ox-LDL, and 34.5 ± 33.4 cpm/mg protein for 40 µg/ml ox-LDL. Measurements of cytotoxicity were performed since toxic effects of ox-LDL towards various cell species have been described. Up to a concentration of 50 µg/ml ox-LDL or native LDL respectively, cell viabilities were > 95 % as measured by trypan blue exclusion. 

### Effects of ox-LDL on arginine transport 

To investigate whether ox-LDL induced inhibition of NO production was associated with a decreased arginine uptake, J774.A1 cells were incubated with [^3^H]L-arginine as described in METHODS. Arginine transport into the cells was not significantly reduced when incubated with increasing amounts (0-50 µg/ml ox-LDL f.c., data not shown). It can be concluded that ox-LDL induced inhibition of inducible NO synthesis is not a consequence of reduced cellular arginine uptake. 

### Effects of ox-LDL on iNOS mRNA expression 

**Figure 2:**
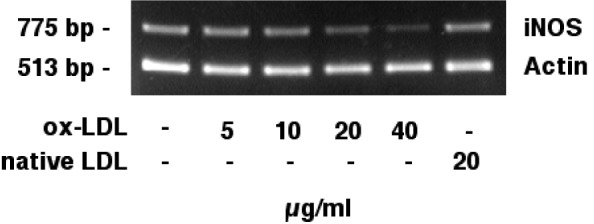
Effects of HOCl-oxidized LDL on iNOS mRNA expression (semiquantitative RT-PCR). J774.A1 mouse macrophages were cultured in lipoprotein-deficient medium for 24 hours followed by an incubation with 0-40 µg/ml HOCl-oxidized LDL or 20 µg/ml native LDL and IFN-gamma/LPS for 24 hours. RNA was extracted and analyzed as described in the METHODS section, single-stranded cDNA synthesis was performed, and DNA was amplified by semiquantitative competitive PCR using specific primers for iNOS and actin. iNOS mRNA (775 bp) showed a dose dependent reduction when cells were incubated with increasing amounts (0-40 µg/ml) of ox-LDL (lanes 1-5). Native LDL (lane 6) did not reduce iNOS mRNA levels. Actin mRNA (513 bp) levels remained unchanged. Data shown are representative for three independent experiments.

Figure 2(Fig. 2) shows the competitive semiquantitative RT-PCR analysis of iNOS mRNA in J774.A1 cells. iNOS mRNA (775 bp) showed a dose dependent reduction when cells were incubated with increasing amounts of ox-LDL (lanes 1-5). Native LDL (20 µg/ml; lane 6) did not reduce iNOS mRNA levels. Actin mRNA (513 bp) levels remained unchanged. Our data show that HOCl-oxidized LDL, but not native LDL, decreases iNOS mRNA levels. 

### Effects of ox-LDL on iNOS protein expression 

**Figure 3:**
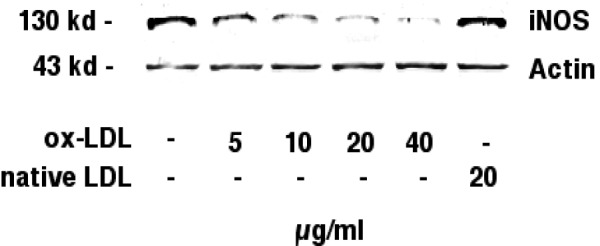
Immunoblotting against inducible nitric oxide synthase. J774.A1 mouse macrophages were cultured in lipoprotein-deficient medium for 24 hours, stimulated with IFN-gamma/LPS, and incubated with increasing amounts (0-40 µg/ml) of HOCl-oxidized LDL or native LDL (20 µg/ml) for 24 hours. Western blotting was performed as described in the METHODS section. Immunoblotting identified a band with an estimated molecular mass of 130 kD, the known molecular mass of inducible nitric oxide synthase, in stimulated J774.A1 mouse macrophages. iNOS protein showed a dose dependent reduction when cells were incubated with increasing amounts (0-40 µg/ml) of ox-LDL. Native LDL did not reduce iNOS protein levels. Actin (43 kD) levels remained unchanged during incubations with ox-LDL. Data shown are representative for three independent experiments.

Figure 3(Fig. 3) shows the Western blot analysis of inducible nitric oxide synthase in J774.A1 cells. IFN-gamma/LPS stimulated J774.A1 mouse macrophages were incubated with with increasing amounts (0-40 µg/ml) of HOCl-oxidized LDL or native LDL (20 µg/ml) for 24 hours. Western blotting was performed as described in the METHODS section. 

### Effects of ox-LDL on macrophage growth 

**Figure 4:**
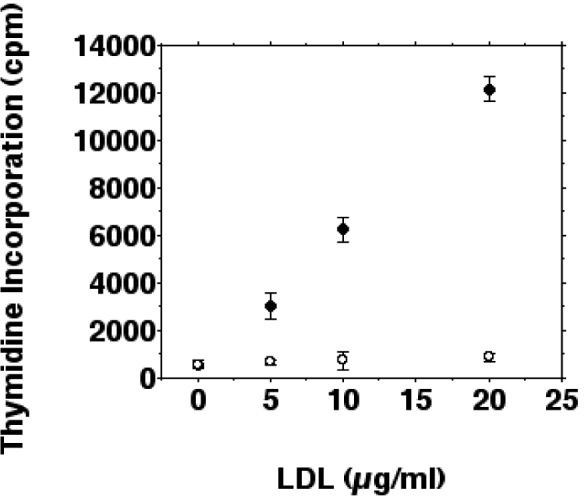
Effects of ox-LDL and native LDL on the proliferation of IFN-gamma/LPS stimulated macrophages. J774.A1 monocyte/macrophages were preincubated with DME medium containing 10 % LPDS for 24 hours. Cells were stimulated with IFN-gamma/LPS and incubated with increasing amounts (0-20 µg/ml f.c.) of oxidized LDL (• ) or native LDL (o) for 24 hours. Cell proliferation was measured as tritiated thymidine ([^3^H]thymidine) incorporation into macrophages as described in METHODS. Data show the mean of triplicate measurements ± SD and are representative of three independent experiments.

Tritiated thymidine incorporation was determined in IFN-gamma/LPS stimulated and unstimulated cells to examine whether inhibition of inducible nitric oxide synthesis by ox-LDL stimulates proliferation of J774.A1 macrophages. iNOS induction by IFN-gamma/LPS reduced basal tritiated thymidine incorporation of J774.A1 cells by more than 95 %. The inhibition of basal tritiated thymidine incorporation was antagonized by ox-LDL. As shown in Figure 4(Fig. 4), tritiated thymidine uptake increased 21.5-fold after a 24 hour incubation with 20 µg/ml ox-LDL for stimulated cells. The increase of tritiated thymidine incorporation was linear in a dose-dependent manner up to 20 µg/ml ox-LDL for IFN-gamma/LPS stimulated macrophages. When incubated with more than 50 µg/ml ox-LDL thymidine incorporation decreased probably due to cytotoxic effects of ox-LDL on macrophages. 

The correlation between inhibition of inducible nitric oxide synthesis by ox-LDL and increased thymidine incorporation was linear from 0-20 µg/ml ox-LDL with a correlation coefficient of 0.98. The slight (1.5-fold) increase of tritiated thymidine incorporation after a 24 hour incubation with native LDL (20 µg/ml) was not statistically significant. Incubations of unstimulated cells with 0-20 µg/ml ox-LDL showed a 2.75-fold increase of tritiated thymidine incorporation after 24 hours (data not shown). These results suggest that the inhibition of inducible nitric oxide synthesis by ox-LDL has the capability to augment growth (i.e., increase in cell number) of IFN-gamma/LPS stimulated J774.A1 macrophages. 

**Figure 5:**
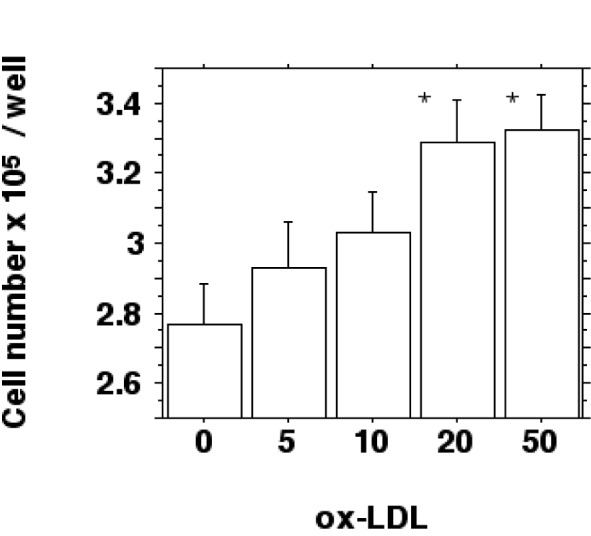
Ox-LDL induced increase of cell number. J774.A1 monocyte/macrophages were preincubated with DME medium containing 10 % LPDS for 24 hours. Cells were stimulated with IFN-gamma/LPS and incubated with increasing amounts (0-50 µg/ml f.c.) of oxidized LDL for 24 hours. Cell counting was performed by phase-contrast microscopy as described in METHODS and is shown as cell number x 10^5^ per well. Data show the mean of triplicate measurements ± SD and are representative of three independent experiments. *P < 0.05 compared to control incubations without ox-LDL.

To prove this notion the number of macrophages per well was determined by inverted phase-contrast microscopy (cell counting). The results show an increased cell proliferation after a 24 hour incubation with 0-50 µg/ml ox-LDL for IFN-gamma/LPS stimulated macrophages (Figure 5(Fig. 5)). 

Cell number increased from 2.78 ± 0.2 x 10^5^ cells/well for control incubations without LDL to 3.29 ± 0.21 x 10^5^ cells/well for 20 µg/ml ox-LDL and 3.32 ± 0.18 x 10^5^ cells/well for 50 µg/ml ox-LDL (*P *< 0.05). Native LDL had no significant effect on cell number (2.83 ± 0.21 x 10^5^ cells/well for control incubations without LDL against 2.76 ± 0.12 x 10^5^ cells/well for 20 µg/ml native LDL and 2.82 ± 0.12 x 10^5^ cells/well for 50 µg/ml native LDL; differences not statistically significant). 

To prove that ox-LDL induced proliferation is a consequence of iNOS inhibition, J774.A1 mouse macrophages were seeded at a density of 2 x 10^5^ per well, stimulated with IFN-gamma/LPS, and cultured with HOCl-oxidized LDL (0-50 µg/ml), or with ox-LDL and 300 µM of the nitric oxide synthase inhibitor L-NAME for 24 hours. The addition of L-NAME to stimulated macrophages increased the cell number from 2.01 ± 0.04 x 10^5^ cells/well (control incubations without L-NAME) to 3.12 ± 0.35 x 10^5^ cells/well. This was accompanied by an almost complete inhibition of NO synthesis. The addition of HOCl-oxidized LDL (0-50 µg/ml) to L-NAME containing incubations induced no further statistically significant increase in cell number (data not shown). This shows that ox-LDL induced proliferation of stimulated macrophages is a consequence of iNOS inhibition.

### Effect of nitric oxide on ox-LDL induced macrophage proliferation 

**Figure 6:**
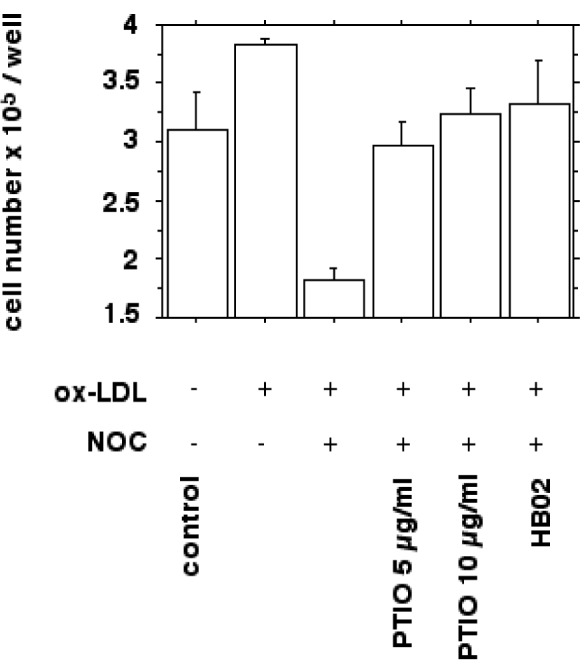
Bar chart showing the effect of nitric oxide donors and ox-LDL on macrophage growth in vitro as quantified by cell counting. Cells were prepared as described in METHODS. IFN-gamma/LPS stimulated J774.A1 mouse macrophages were seeded at a density of 2 x 10^5^ per well and cultured with or without 10 µg/ml HOCl-oxidized LDL for 24 hours. Ox-LDL induced proliferation was inhibited by the NO donor NOC 18 (30 µg/ml; *P*<0.0001). The inhibition of cell proliferation could be antagonized by adding NO scavengers carboxy-PTIO (10 and 20 µg/ml; *P*<0.005) and oxygenated hemoglobin (100 µM HbO_2_; *P*<0.005). Values are given as cell number x 10^5^ per well. Error bars represent SD of triplicate measurements.

We next examined whether the increased macrophage proliferation could be antagonized by the addition of nitric oxide donors (NOC 18) and if the antiproliferative actions of nitric oxide could be reversed by nitric oxide scavengers (carboxy-PTIO, hemoglobin). Figure 6(Fig. 6) shows the increased proliferation of IFN-gamma/LPS stimulated macrophages by 10 µg/ml ox-LDL in vitro as quantified by cell counting after a 24 hours incubation. 

In addition, measurements of thymidine incorporation and cell protein were performed as described in METHODS. Correlation between the means of thymidine incorporation and cell count was linear with a correlation coefficient of 0.92. Correlation between the means of cell protein and cell count was linear with r = 0.93.

### Effects of ox-LDL and nitric oxide on macrophage apoptosis 

NO mediated apoptosis has been described for various cell lines (Cheng et al. 2014[8]). To test if ox-LDL mediated inhibition of inducible nitric oxide synthesis prevents apoptosis in J774.A1 macrophages we assessed cytosolic DNA fragments as described in METHODS. 

**Figure 7:**
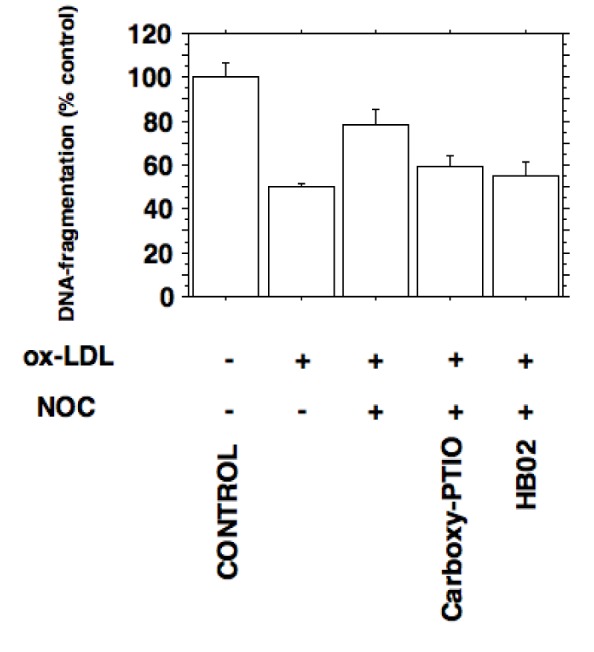
Inhibition of inducible nitric oxide synthesis by ox-LDL prevents apoptosis in J774.A1 macrophages. Apoptosis was quantified by detection of histone-associated-DNA-fragments as described in METHODS. Stimulated J774.A1 mouse macrophages were cultured in lipoprotein-deficient medium for 24 hours followed by an incubation with or without 10 µg/ml HOCl-oxidized LDL for 24 hours. Ox-LDL inhibits the apoptosis of macrophages expressing iNOS activity (*P *< 0.0005). Decreased DNA-fragmentation by ox-LDL was increased by the NO donor NOC 18 (30 µg/ml; *P *< 0.005). The nitric oxide mediated increase in apoptosis could be antagonized by adding the NO scavengers carboxy-PTIO (20 µg/ml; *P *< 0.02) and oxygenated hemoglobin (100 µM HbO2; *P *< 0.02). Error bars represent SD.

The decrease of cytosolic DNA fragments in stimulated macrophages incubated with ox-LDL shows that the proliferative actions of ox-LDL are associated with a decrease of NO-induced apoptosis (Figure 7(Fig. 7)). 

## Discussion

The present study shows a correlation between macrophage growth and the inhibition of inducible nitric oxide synthase by ox-LDL. The inhibition of iNOS by copper-oxidized LDL has been demonstrated (Matsuno et al., 1997[31]; Yang et al. 1994[46]). Oxidative modification of LDL results in the formation of lipid hydroperoxides and promotes atherosclerosis (Fan et al., 2014[14]). The inhibition of iNOS by the lipid hydroperoxide 13-hydroperoxyl octadecadienoic (13-HPODE) acid has been previously described (Huang et al., 1999[24]). During our experiments hypochlorite (HOCl) oxidized LDL was used because its physiological relevance has been emphasized (Hazell et al., 1996[20]), and the occurrence of copper-oxidized LDL in vivo has not yet been confirmed. HOCl is an oxidant produced from H_2_O_2_ and chloride by myeloperoxidase (MPO, E.C. 1.11.1.7) in vivo. MPO is present in its active form in human atherosclerotic lesions (Daugherty et al., 1994[10]). HOCl aggregates and transforms LDL into a high-uptake form for macrophages in vitro. Using monoclonal antibodies HOCl-oxidized proteins have been demonstrated in human atherosclerotic lesions and were predominantly found in monocyte/macrophages, smooth muscle cells, and endothelial cells (Hazell et al., 1996[20]). Proliferation of macrophages within atherosclerotic lesions has been shown (Robbins et al., 2013[35]), and in situ hybridisation confirmed the presence of iNOS in macrophages, foam cells, and vascular smooth muscle cells of atherosclerotic vessels (Buttery et al., 1996[7]). On the basis of a recent report showing a lower percentage of proliferating macrophages in early plaques compared with advanced plaques, it seems to be variable in different stages of the disease (Robbins et al., 2013[35]). 

Contradictory results have been reported regarding an ox-LDL induced iNOS inhibition and a decrease of iNOS mRNA. Both transcriptional mechanisms with an ox-LDL associated decrease of iNOS mRNA synthesis (Hamilton et al., 1995[18]; Huang et al., 1999[24]) and posttranscriptional mechanisms of ox-LDL induced iNOS inhibition without significant effects of ox-LDL towards iNOS mRNA levels (Wu et al., 1998[42]; Yang et al. 1994[46]) have been postulated. According to our data, iNOS mRNA is reduced in a dose dependent manner after an incubation of J774.A1 mouse macrophages with increasing amounts (0-40 µg/ml) of HOCl-oxidized LDL. In contrast, native LDL did not reduce iNOS mRNA levels. Actin mRNA levels remained unchanged, thereby excluding toxic effects of HOCl-oxidized LDL. The decrease of iNOS mRNA was associated with a dose-dependent decrease of iNOS protein, as revealed by immunoblotting. 

Formation of nitric oxide by the constitutive NOS expressed in endothelial cells is believed to protect against the development of atherosclerotic lesions. Under conditions with decreased constitutive NOS activity, iNOS might substitute the synthesis of NO (Kanno et al., 2000[26]; Yan et al., 1996[45]). iNOS expression within macrophages of atherosclerotic lesions has been described. Previous studies were conducted on normal and atherosclerotic human vessels by in situ hybridisation and immuno-histochemistry using probes for iNOS. Inducible NOS was not detected in normal vessels but widespread iNOS protein staining was found in early and advanced lesions in macrophages, foam cells, and vascular smooth muscle cells of atherosclerotic vessels (Baker et al., 1999[4]; Buttery et al., 1996[7]). Inhibition of iNOS by ox-LDL might contribute to the development of atherosclerotic plaques by an increased proliferation of macrophages due to the diminished release of antiproliferative nitric oxide in these lesions.

iNOS expression in vascular smooth muscle cells and macrophages may be beneficial as a compensatory mechanism for the lack of endothelial NO synthesis (Kanno et al., 2000[26]; Yan et al., 1996[45]), thereby preventing restenosis following angioplasty or heart transplant vasculopathy (Hecker et al., 1999[21]). This idea is supported by a paper, showing that the administration of an iNOS selective inhibitor L-N^6^-(1-iminoethyl)-lysine accelerated the progression of vasculopathy in transplantation atherosclerosis, and transduction with iNOS using an adenoviral vector has been shown to completely suppress the development of allograft arteriosclerosis (Forbes et al., 2013[15]; Shears et al., 1997[37]). Induction of iNOS leads to a reduced proliferation in both medial and intimal smooth muscle cells (Yan and Hansson, 1998[44]) and iNOS was shown to restore blood flow in the injured artery after denudation of the rat carotid artery as a model for arterial injury and restenosis (Yan et al., 1996[45]). iNOS gene transfer to rats and pigs has been shown to inhibit intimal hyperplasia in response to vascular injury in vivo. This protective effect was reversed by selective iNOS inhibition (De Meyer et al., 2000[12]; Shears et al., 1998[38]). A higher incidence of atherosclerotic plaques, an elevated systolic blood pressure, and increased serum cholesterol levels have been described for iNOS deficient mice (Ihrig et al., 2001[25]). Immunohistochemical evaluations of atherosclerotic lesions from iNOS knockout mice have shown activated endothelial cells and lipid-loaden macrophages (foam cells), and oxidized low-density lipoprotein immune complexes, suggesting that advanced vascular disease in iNOS knockout mice can be mediated by increased accumulation of oxLDL immune complexes (Al Gadban et al., 2012[1]). According to these results, inducible nitric oxide synthesis can be supposed to be a compensatory mechanism for the lack of endothelial NO formation, thereby maintaining vascular homeostasis. 

Myogenic and macrophagic foam cells are commonly detected in experimental and human fibro-atheromatous plaques. In a thymidine-autoradiographic study combined with ultrastructural observations in the human fibro-atheromatous plaques most of the autoradiographic silver grains appeared on foam cells and monocyte-like cells, thus suggesting a local proliferation of these cells (Villaschi and Spagnoli, 1983[40]). Simultaneous thymidine autoradiography and immunostaining with cell type-specific monoclonal antibodies revealed that approximately 30 % of the labeled cells were macrophages and 45 % were smooth muscle cells in advanced atherosclerotic lesions (Rosenfeld and Ross, 1990[36]). Increased macrophage proliferation resulting from a decrease of iNOS dependent nitric oxide production might enhance the development of atherosclerotic lesions. 

Cytokines such as IFN-gamma have previously been shown to inhibit DNA synthesis, cell growth and division by a NO-dependent mechanism (Garg and Hassid, 1989[16]). In addition, induction of iNOS by IFN-gamma and LPS was associated with an increase in apoptosis of murine peritoneal macrophages (Albina et al., 1993[2]). Nitric oxide synthesis by iNOS correlates inversely with macrophage life span in vitro. It has been proposed that NO-dependent death of murine peritoneal macrophages activated in vitro with IFN-gamma and LPS is mediated through apoptosis. This was shown by microscopic examination of the cells, which revealed the presence of nuclear and cytoplasmic alterations characteristic of apoptosis, and by the specific pattern of internucleosomal DNA fragmentation detected by electrophoresis (Albina et al., 1993[2]). Nitric oxide induced apoptosis is presumed to be due to cyclic GMP dependent protein kinase G activation (Loweth et al., 1997[29]). Inhibition of NO formation by N-monomethyl-L-arginine (L-NMA) diminished apoptosis of IFN-gamma/LPS activated J774.A1 macrophages (Yang et al., 1996[47]). Our data indicate that inhibition of iNOS dependent nitric oxide production by oxidized LDL enhances macrophage proliferation and is associated with diminished NO-mediated apoptosis. 

Inducible nitric oxide synthesis blocks cell proliferation via a cGMP dependent pathway (Shears et al., 1998[38]), and independent of cGMP through the p42/44 mitogen-activated protein kinase (MAPK) signaling cascade with upregulation of the cell cycle inhibitor p21 (Kibbe et al., 2000[27]). In addition to a local proliferation, the appearance of macrophages within atherosclerotic lesions has been supposed to be the consequence of an increased recruitment of monocytes via NF-kappa B dependent expression of vascular endothelial cell adhesion-molecule 1 (VCAM-1), macrophage-colony-stimulating-factor (M-CSF), and monocyte chemoattractant protein 1 (MCP-1) by human vascular endothelial cells. NO has been shown to inhibit VCAM-1 (De Caterina et al., 1995[11]), M-CSF (Peng et al., 1995[34]), and MCP-1 expression (Zeiher et al., 1995[49]) by inhibiting NF-kappa B activation. Inhibition of NO synthesis by L-NMA was shown to activate NF-kappa B with concomitant VCAM-1, M-CSF, and MCP-1 expression (De Caterina et al. 1995[11]; Peng et al. 1995[34]; Zeiher et al. 1995[49]). Inhibition of inducible nitric oxide synthesis by ox-LDL might influence macrophage accumulation within atherosclerotic lesions by enhancing NF-kappa B dependent VCAM-1, M-CSF, and MCP-1 expression. 

Numerous anti-atherogenic properties of nitric oxide in vitro have been described. NO inhibits LDL oxidation (Hogg et al., 1995[23]), smooth muscle cell proliferation (Garg and Hassid, 1989[16]), smooth muscle cell migration (Dubey et al., 1995[13]), neutrophil adhesion (Lefer and Ma, 1993[28]), MCP-1 expression (Zeiher et al., 1995[49]), and inhibits NF-kappa B activation (Peng et al., 1995[34]; Zeiher et al., 1995[49]). Using IFN-gamma/LPS stimulated macrophages we examined the influence of iNOS antagonists (L-NAME), nitric oxide donors (NOC 18), and nitric oxide scavengers (carboxy-PTIO, hemoglobin) on ox-LDL induced macrophage proliferation. Our results contribute to the concept that inducible NO release can protect against atherogenesis by preventing macrophage proliferation and ox-LDL might contribute to the development of atherosclerotic lesions by reducing inducible nitric oxide synthesis with concomitant macrophage proliferation. 

## Acknowledgements

We thank Mrs. Inge Pichler for excellent technical assistance. The work was funded by the regular budget of the Medical University Vienna. The authors declare that they have no conflict of interest.
